# Computer Game Use and Television Viewing Increased Risk for Overweight among Low Activity Girls: Fourth Thai National Health Examination Survey 2008-2009

**DOI:** 10.1155/2014/364702

**Published:** 2014-06-05

**Authors:** Ladda Mo-suwan, Jiraluck Nontarak, Wichai Aekplakorn, Warapone Satheannoppakao

**Affiliations:** ^1^Department of Pediatrics, Faculty of Medicine, Prince of Songkla University, Songkhla 90110, Thailand; ^2^Office of National Health Examination Survey, Health System Research Institute, Bangkok 11000, Thailand; ^3^Department of Community Medicine, Faculty of Medicine Ramathibodi Hospital, Mahidol University, Bangkok 10400, Thailand; ^4^Faculty of Public Health, Mahidol University, Bangkok 10400, Thailand

## Abstract

Studies of the relationship between sedentary behaviors and overweight among children and adolescents show mixed results. The fourth Thai National Health Examination Survey data collected between 2008 and 2009 were used to explore this association in 5,999 children aged 6 to 14 years. The prevalence of overweight defined by the age- and gender-specific body mass index cut-points of the International Obesity Task Force was 16%. Using multiple logistic regression, computer game use for more than 1 hour a day was found to be associated with an increased risk of overweight (adjusted odds ratio (AOR) = 1.4; 95% confidence interval: 1.02–1.93). The effect of computer game use and TV viewing on the risk for overweight was significantly pronounced among girls who spent ≤3 days/week in 60 minutes of moderate-intensity physical activity (AOR = 1.99 and 1.72, resp.). On the contrary, these sedentary behaviors did not exert significant risk for overweight among boys. The moderating effect on risk of overweight by physical inactivity and media use should be taken into consideration in designing the interventions for overweight control in children and adolescents. Tracking societal changes is essential for identification of potential areas for targeted interventions.

## 1. Introduction 

Childhood obesity has emerged as a significant health problem in a transitional society. Rapidly changing dietary practices and a sedentary lifestyle have led to a high prevalence of childhood overweight and obesity among school-aged children (defined by the International Obesity Task Force cut-points) in developing countries between 2004 and 2010: 41.8% in Mexico, 22.1% in Brazil, 13.3–22.3% in South Africa, 27.9% in Argentina, and 2.8–28% in India [1]. In Thailand, data from the two national surveys demonstrated an increase of the obesity prevalence among the 6- to 12-year-old children from 5.8% in 1997 to 6.7% in 2001 by using the weight-for-height criteria of a local reference [2]. This rising trend coincided with an increase of type 2 diabetes among Thai diabetic pediatric patients from 5% between 1986 and 1995 to 17.9% between 1996 and 1999 [3].

Increased energy content in diet, decreased levels of physical activity, and increased sedentary lifestyles as well as a number of cultural and environmental factors have been identified as the causes of obesity in children [4–6]. In the Thai context, determinants of overweight and obesity among children in previous reports included having obese parents, being in a family with a high income, maternal overweight prior to pregnancy, high birth weight, being the only child, large amounts of food consumed by children, having a lesser amount of exercise than peers, and TV viewing time more than 2 hours per day [7–10].

Changes in several social and environmental factors have been suggested as causes of the “obesity epidemic” among children, for example, reduced physical education at school, increased homework loads, school vending machines, TV, larger food portion sizes, fast-food restaurants, video games, and many others [11]. Time spent in seated sedentary behaviors (SB) (e.g., electronic media use) reduces time allocation for physical activity (PA) and hence increases risk of overweight and obesity. Moreover, snack and sugar-sweetened beverage consumption while watching TV further augments the positive energy balance. Children worldwide have increasing access to electronic media, such as TV, computer/video games, cell phones, and the internet in daily life. In Thailand, the number of internet cafés which provide online game service increased 1.8 times over 2 years from 2008 to 2010 [12]. The fourth National Health Examination Survey in Thailand revealed that 5% of the 6 to 9 year olds and 12% of the 10 to 14 year olds engaged in computer games more than 1 hour each day, while 57.1% and 73.1%, respectively, watched TV more than 2 hours/day during the weekdays [13]. A report from the Child and Adolescent Mental Health Center in Bangkok demonstrated an increase in the game addiction rate from 5% in 2005 to 9% in 2009 [12]. In regard to media use, an association of sedentary behavior, primarily through TV viewing, with body mass index (BMI) and obesity has been well documented [14, 15], while the relationship of seated computer game use with obesity was unclear. Most of the studies investigated total screen time including TV viewing, working on a computer, and playing video games [15, 16]. A limited number of studies that looked at computer games independently had mixed results [16–19].

In order to gain an understanding of the current rising prevalence of childhood overweight, we used recent data from the fourth National Health Examination Survey (NHES IV) to investigate the associations of SB (i.e., time spent at computer gaming and television viewing) with overweight among children aged 6 to 14 years in Thailand. In addition, we examined whether the effect of screen time on overweight varied by physical activity status and gender.

## 2. Subjects and Methods

### 2.1. Design

The National Health Examination Surveys have been conducted every 5 years since 1991. The fourth National Health Examination Survey (NHES IV) 2008-2009 conducted by the National Health Examination Survey Office was designed to represent the noninstitutionalized Thai population using a multistage stratified sampling based on 2008 Thai population registers [20]. For the first stage, 5 provinces were randomly sampled by proportion to size (PPS) from each of the 4 regions, except Bangkok. In the second stage, 3 to 5 districts were selected by PPS from each province. In the third stage, in each province, 13-14 electoral units (EUs) or villages were selected by PPS from each of the urban and rural areas. In the final stage, for each EU/village, 8 to 10 males and 8 to 10 females were selected by systematic random sampling from population registers from each of the six broad age and gender groups (1–14-, 15–59-, and ≥60-year-old males/females). In Bangkok, 5 to 6 EUs were randomly selected by PPS from each of the 12 districts. The final stage was identical to the methods used in other provinces.

### 2.2. Subjects

The NHES IV enrolled 29,485 subjects aged 1 to 60+ years. The final sample size of the 1- to 14-year-old subjects was 9,035 individuals (response rate of 92.8%). The response rate for boys and girls was 92.4% and 93.1%, respectively. This report presents an analysis of the data from the school-aged population: 5,999 children aged 6 to 14 years.

### 2.3. Data Collection

Subjects were weighed in light clothes using a Tanita scale and recorded to the nearest 0.1 kg. Standing heights were measured by trained research assistants using a locally made stadiometer and recorded to the nearest 0.1 cm.

Demographic and socioeconomic data were obtained by interviewing the parents. Information regarding dietary intake and physical and sedentary activities was taken by interviewing the parents of children under 10 years of age and the subjects themselves for those aged 10 years and older. The questions for media use were “How many hours a day during the past month did you watch television?” and “How many hours a day during the past month did you play computer games?” For physical activity, the question was “How many days during the past week did you have physical activity that increased your breathing rate and heart rate for at least 60 minutes?”

### 2.4. Statistical Analysis

Overweight was defined using the age- and gender-specific BMI cut-points of the International Obesity Task Force [21]. For media use, TV time was categorized into 2 hours/day or less and more than 2 hours/day according to the American Academy of Pediatrics recommendation [22] and time spent in playing computer games was classified into 1 hour/day or less and more than 1 hour/day. Exercise of moderate intensity was grouped into having 60 minutes for 3 days/week or more and less than 3 days/week. Since gender difference has been noted in the studies of obesity especially in relation to physical activity behaviors [15, 23], the analysis was thus computed separately for boys and girls. Distribution of all study variables was explored by descriptive statistics. Logistic regressions were computed to examine the association of media use and physical activity level with overweight. Consumption of high energy snacks (examples given were potato crisps and rice-shrimp crisps), which was highly associated with overweight in both male and female subjects, was selected as a covariate in the logistic regression analysis. In addition, family income, which was reported to be associated with overweight in Thai children, [7] was also included in the regression models. The analyses were performed using STATA 11.0 [24].

### 2.5. Ethical Clearance

The National Ethical Review Committee for Research in Human Subjects, Ministry of Public Health, approved the study. The sampled families were informed of the data collection process and verbal permission was obtained.

## 3. Results

Table 1 describes overweight prevalence, media use, physical activity, consumption of high energy snack, and family income characteristics of the subjects. Prevalence of overweight using age- and gender-specific BMI cut-points of the International Obesity Task Force [21] was 15.2% for girls versus 16.7% for boys. Most subjects watched TV more than 2 hours/day. On the other hand, only 5.2% engaged in computer games more than 1 hour/day; male subjects played computer games twice as much as the females (*P* < 0.001). Male subjects also engaged in moderate-intensity physical activity significantly more than the females (*P* < 0.001). No gender difference was noted in the frequency of having high energy snacks and family income levels.

The associations of media use and physical activity with overweight are shown in Table 2. From the multiple logistic regression analysis, computer game use and physical activity behavior had a significant effect on the likelihood of overweight among the 6- to 14-year-old subjects. With the 1 hour/day or less as the referent group, use of computer games more than 1 hour/day increased the likelihood of overweight (adjusted odds ratio (AOR) = 1.40; 95% confidence interval (CI): 1.02–1.93), whereas children who engaged in 60 minutes of moderate-intensity physical activity for more than 3 days/week were less likely to be overweight (AOR = 0.75; 95% CI: 0.66–0.84). The subgroup analysis by gender shows different risks between boys and girls. Watching TV more than 2 hours/day significantly increased the risk for overweight (AOR = 1.67; 95% CI: 1.17–2.40), while engagement in moderate-intensity physical activity significantly decreased the risk among girls (AOR = 0.62; 95% CI: 0.51–0.76). For boys, associations of computer game use and engagement in moderate-intensity PA with overweight were no longer significant after adjustment for high energy snack consumption and family income.

The relationship between media use and overweight was further explored by a subgroup analysis according to the PA level (Table 3). The effect of media use was significant only among the girls with a lower level of PA. Female subjects who spent ≤3 days/week in 60 minutes of moderate-intensity PA had a greater risk for overweight if they spent more than 2 hours/day viewing TV (AOR = 1.72; 95% CI: 1.09–2.74 versus 2 hours/day or less) or more than 1 hour/day playing computer games (AOR = 1.99; 95% CI: 1.15–3.47 versus 1 hour/day or less), while those having more than 3 days/week of moderate-intensity PA and engagement in either TV viewing or computer gaming did not pose significant risks. For boys, media use did not exert significant risk for overweight in either group of PA.

## 4. Discussion

By using the data from the recent National Health Examination Survey, we found that computer game use for more than 1 hour a day was associated with an increased risk for overweight while engagement in moderate-intensity PA significantly decreased the likelihood of overweight among the 6- to 14-year-old female subjects. Further analysis showed that the effect of computer game use and TV viewing on the risk for overweight was significant among girls, but not boys, who spent ≤3 days/week in 60 minutes of moderate-intensity PA.

The association between the level of PA and weight status has been shown to be nonuniform in the recent systematic review of cross-sectional studies [15]. Like ours, most of the reports that found significant negative associations, the outcomes were different for boys and girls. Gender differences in health behaviors relating to obesity have been documented in other studies [23, 25, 26]. These results may be due to the differences in physiological responses or gender role expectation in the society for boys and girls [27–29]. On the other hand, child weight status itself may influence the intensity and frequency of PA and this may give rise to the mixed findings of their associations [15]. Moreover, the self-report of PA which was used in most surveys may affect the accuracy and lead to inconsistent results.

The positive association between sedentary behaviors, namely, TV viewing or playing video/computer games, and child weight status found in this study is consistent with the majority of previous studies in the recent systematic review [15]. Research on the association between screen time and overweight/obesity in children mostly used total screen time (i.e., combining TV viewing, video/computer game use, and non-leisure computer use). Nowadays, the media such as computer games and the internet substantially occupies children's pastime. An investigation of the independent effect of this electronic media use on overweight/obesity in children will have public health importance. Like studies of physical activity, research assessing the effect of video/computer game use on the weight status of children showed mixed results. A meta-analysis by Marshall et al. [16] and three other recent studies [30–32] among children and adolescents found no association, while a positive relationship between electronic game use and weight status were found to be curvilinear with a difference in ages in a study of US children using 24-hour time-use diaries to record the amount of video game use [19]. Children under the age of 8 with higher weight status played moderate amounts of electronic games, while children with lower weight status played either very little or a lot of electronic games. This study also found that girls, but not boys, with higher weight status played more video games. Video game use was also found to be associated with an increased cardiometabolic risk score, blood pressure, and lipids [17, 18]. As children usually engage in multiple SB, an effect of each activity on child weight status should be disentangled to provide evidence for development of effective interventions to promote healthy behaviors for obesity control.

We found physical activity to be a moderator of the relation between sedentary activities (i.e., TV viewing time and computer game time) and overweight. Girls who had 60 minutes of moderate-intensity PA ≤ 3 days/week were more likely to be at risk for overweight if they watched TV > 2 hours/day or played computer games > 1 hour/day than those who had such level of PA > 3 days/week. This moderating effect was not observed in boys. In a study of 9,278 Taiwanese adolescents, Yen et al. demonstrated that the relationship between increased BMI and a high level of TV viewing was found only in adolescents who exercised less than 1 hour/day, but not in those who exercised 1 hour/day or more [33]. These findings indicated that adequate exercise may be protective for children who spend a lot of time on sedentary activities from increased adiposity, particularly among girls. These moderators, namely, age, gender, and PA, should be taken into account in developing interventions for children and adolescents. However, in the present study, it is not clear whether the findings of differences in the associations by gender are due to a variation in energy expenditures pattern during TV watching or in the type of computer game use between boys and girls. The reasons behind this discrepancy need further investigation.

Our study has some limitations. As is the nature of surveys, one could not interpret the findings as causal. The PA and SB were collected by questionnaires. Given the publicized untoward effect of TV viewing and computer game use, the bias may be likely towards underreporting. Nevertheless, this study has its strength as a national representative study with a relatively large sample size. The findings could reflect the behavioral pattern of Thai children and a meaningful relationship of these behaviors to child overweight problem.

## 5. Conclusions

The lives of children nowadays have been considerably affected by societal changes and media technologies. We found that computer game use for more than 1 hour a day and TV viewing for more than 2 hours a day increased the risk for overweight among girls who had a low PA level. These findings are helpful for developing effective interventions to promote healthy behaviors for the control of obesity. As computers are being used in schools and at home for educating children at a younger age, the risk of this electronic media should be communicated to the families and the public as well. Parents should be informed to discipline the use of a computer in order to control sedentary time. Tracking societal changes on lifestyles should be carried out regularly to identify potential areas for targeted interventions.

## Figures and Tables

**Table 1:** Subject characteristics.

Characteristics	Total *N* = 5,998	Boys *n* = 2,972	Girls *n* = 3,026
Overweight (%, 95% CI)	16.0 (14.6–17.4)	16.7 (15.0–18.5)	15.2 (13.7–16.9)
Watching TV (%, 95% CI)	*N* = 5,778	*n* = 2,854	*n* =2,924
More than 2 h/d	89.3 (88.1–90.3)	88.2 (86.8–89.5)	90.4 (88.8–91.8)
Computer game use (%, 95% CI)	*N* = 5,777	*n* = 2,855	*n* = 2,922
More than 1 h/d	5.2 (4.4–6.2)	7.1 (6.0–8.5)^#^	3.2 (2.5–4.1)^#^
Having 60 minutes of moderate-intensity physical activity (%, 95% CI)	*N* = 5,925	*n* = 2,934	*n* = 2,991
More than 3 d/wk	41.6 (38.4–44.9)	48.6 (44.6–52.7)^#^	34.4 (31.8–37.1)^#^
Frequency of high energy snack consumption (%, 95% CI)	*N* = 5,985	*n* = 2,964	*n* = 3,021
1 time/d or more	4.2 (3.5–4.9)	3.7 (2.9–4.7)	4.6 (4.0–5.3)
Family income (%, 95% CI)	*N* = 5,374	*n* = 2,671	*n* = 2,703
15,000 B/month or more	24.4 (21.5–27.5)	25.4 (22.5–28.6)	23.3 (20.1–26.8)

CI: confidence interval; ^#^ *P* < 0.001.

**Table 2:** Odds ratio for the associations of computer game use and television viewing with overweight by gender.

Variables	All		Boys		Girls	
	Unadjusted OR (95% CI)	Adjusted OR* (95% CI)	Unadjusted OR (95% CI)	Adjusted OR* (95% CI)	Unadjusted OR (95% CI)	Adjusted OR* (95% CI)
Computer game use						
1 h/d or less	1	1	1	1	1	1
More than 1 h/d	1.53 (1.13–2.06)	1.40 (1.02–1.93)	1.50 (1.11–2.03)	1.27 (0.92–1.76)	1.50 (0.91–2.45)	1.54 (0.95–2.49)
Watching TV						
2 h/d or less	1	1	1	1	1	1
More than 2 h/d	1.09 (0.88–1.35)	1.11 (0.89–1.39)	0.86 (0.66–1.12)	0.89 (0.68–1.16)	1.64 (1.17–2.30)	1.67 (1.17–2.40)
Having 60 minutes of moderate-intensity physical activity						
3 d/wk or less	1	1	1	1	1	1
More than 3 d/wk	0.71 (0.64–0.80)	0.75 (0.66–0.84)	0.78 (0.64–0.95)	0.85 (0.69–1.03)	0.60 (0.50–0.72)	0.62 (0.51–0.76)

CI: confidence interval. *Adjusted for high energy snack consumption and family income.

**Table 3:** Adjusted odds ratio for associations of computer game use and television viewing with overweight by physical activity levels and gender*.

	Having 60 minutes of moderate intensity physical activity	
	3 days per week or less	More than 3 days per week
	Adjusted odds ratio (95% CI)	Adjusted odds ratio (95% CI)
Boy		
Computer game use		
1 h/d or less	1	1
More than 1 h/d	1.11 (0.70–1.74)	1.36 (0.80–2.31)
Watching TV		
2 h/d or less	1	1
More than 2 h/d	0.93 (0.66–1.32)	0.82 (0.47–1.45)
Girl		
Computer game use		
1 h/d or less	1	1
More than 1 h/d	1.99 (1.15–3.47)	0.77 (0.33–1.80)
Watching TV		
2 h/d or less	1	1
More than 2 h/d	1.72 (1.09–2.74)	1.54 (0.81–2.90)

CI: confidence interval. *Adjusted for high energy snack consumption and family income.

## References

[1] Gupta N, Goel K, Shah P, Misra A (2012). Childhood obesity in developing countries: epidemiology, determinants, and prevention. *Endocrine Reviews*.

[2] Aekplakorn W, Mo-suwan L (2009). Prevalence of obesity in Thailand. *Obesity Reviews*.

[3] Likitmaskul S, Kiattisathavee P, Chaichanwatanakul K, Punnakanta L, Angsusingha K, Tuchinda C (2003). Increasing prevalence of type 2 diabetes mellitus in Thai children and adolescents associated with increasing prevalence of obesity. *Journal of Pediatric Endocrinology and Metabolism*.

[4] Eisenmann JC (2006). Insight into the causes of the recent secular trend in pediatric obesity: common sense does not always prevail for complex, multi-factorial phenotypes. *Preventive Medicine*.

[5] Maffeis C (2000). Aetiology of overweight and obesity in children and adolescents. *European Journal of Pediatrics*.

[6] Taylor AW, Winefield H, Kettler L, Roberts R, Gill TK (2012). A population study of 5 to 15 year olds: full time maternal employment not associated with high BMI. The importance of screen-based activity, reading for pleasure and sleep duration in children’s BMI. *Maternal and Child Health Journal*.

[7] Mo-suwan L, Geater A (1996). Risk factors for childhood obesity in a transitional society in Thailand. *International Journal of Obesity*.

[8] Mo-suwan L, Tongkumchum P, Puetpaiboon A (2000). Determinants of overweight tracking from childhood to adolescence: a 5 y follow-up study of Hat Yai schoolchildren. *International Journal of Obesity*.

[9] Yamborisut U, Kosulwat V, Chittchang U, Wimonpeerapattana W, Suthutvoravut U (2006). Factors associated with dual form of malnutrition in school children in Nakhon Pathom and Bangkok. *Journal of the Medical Association of Thailand*.

[10] Ruangdaraganon N, Kotchabhakdi N, Udomsubpayakul U, Kunanusont C, Suriyawongpaisal P (2002). The association between television viewing and childhood obesity: a national survey in Thailand. *Journal of the Medical Association of Thailand*.

[11] Sturm R (2005). Childhood obesity—what we can learn from existing data on societal trends, part 1. *Preventing Chronic Disease*.

[12] Thailand's Ministry of Culture Situational analysis of game addiction in Thai children. http://www.healthygamer.net/sites/default/files/scribd/game_addiction_2012.pdf.

[13] Mo-suwan L, Aekplakorn W (2011). Health behaviors. *Report of the Fourth National Health Examination Survey 2008-2009: Child Health*.

[14] Tremblay MS, LeBlanc AG, Kho ME (2011). Systematic review of sedentary behaviour and health indicators in school-aged children and youth. *International Journal of Behavioral Nutrition and Physical Activity*.

[15] Prentice-Dunn H, Prentice-Dunn S (2012). Physical activity, sedentary behavior, and childhood obesity: a review of cross-sectional studies. *Psychology, Health & Medicine*.

[16] Marshall SJ, Biddle SJH, Gorely T, Cameron N, Murdey I (2004). Relationships between media use, body fatness and physical activity in children and youth: a meta-analysis. *International Journal of Obesity*.

[17] Goldfield GS, Kenny GP, Hadjiyannakis S (2011). Video game playing is independently associated with blood pressure and lipids in overweight and obese adolescents. *PLoS ONE*.

[18] Saunders TJ, Tremblay MS, Mathieu ME (2013). Associations of sedentary behavior, sedentary bouts and breaks in sedentary time with cardiometabolic risk in children with a family history of obesity. *PLoS ONE*.

[19] Vandewater EA, Shim MS, Caplovitz AG (2004). Linking obesity and activity level with children’s television and video game use. *Journal of Adolescence*.

[20] Aekplakorn W, Chariyalertsak S, Kessomboon P (2011). Prevalence and management of diabetes and metabolic risk factors in Thai adults: the Thai National Health Examination Survey IV, 2009. *Diabetes Care*.

[21] Cole TJ, Bellizzi MC, Flegal KM, Dietz WH (2000). Establishing a standard definition for child overweight and obesity worldwide: international survey. *British Medical Journal*.

[22] American Academy of Pediatrics, Committee on Public Education (2001). American Academy of Pediatrics: Children, adolescents, and television. *Pediatrics*.

[23] Simen-Kapeu A, Veugelers PJ (2010). Should public health interventions aimed at reducing childhood overweight and obesity be gender-focused?. *BMC Public Health*.

[24] StataCorp (2007). *Stata Statistical Software: Release 10*.

[25] Ferrante M, Fiore M, Sciacca GE (2010). The role of weight status, gender and self-esteem in following a diet among middle-school children in Sicily (Italy). *BMC Public Health*.

[26] Govindan M, Gurm R, Mohan S (2013). Gender differences in physiologic markers and health behaviors associated with childhood obesity. *Pediatrics*.

[27] Bird CE, Rieker PP (1999). Gender matters: an integrated model for understanding men’s and women’s health. *Social Science & Medicine*.

[28] Krieger N (2003). Genders, sexes, and health: what are the connections—and why does it matter?. *International Journal of Epidemiology*.

[29] Chiu YF, Chuang LM, Kao HY (2010). Sex-specific genetic architecture of human fatness in Chinese: the SAPPHIRe Study. *Human Genetics*.

[30] Arango CM, Parra DC, Gómez LF, Lema L, Lobelo F, Ekelund U (2013). Screen time, cardiorespiratory fitness and adiposity among school-age children from Monteria, Colombia. *Journal of Science and Medicine in Sport*.

[31] Bickham DS, Blood EA, Walls CE, Shrier LA, Rich M (2013). Characteristics of screen media use associated with higher BMI in young adolescents. *Pediatrics*.

[32] Sisson SB, Broyles ST, Baker BL, Katzmarzyk PT (2011). Television, reading, and computer time: correlates of school-day leisure-time sedentary behavior and relationship with overweight in children in the U.S.. *Journal of Physical Activity & Health*.

[33] Yen CF, Hsiao RC, Ko CH (2010). The relationships between body mass index and television viewing, internet use and cellular phone use: the moderating effects of socio-demographic characteristics and exercise. *International Journal of Eating Disorders*.

